# Composite Tumor of the Larynx: A Case Report

**DOI:** 10.31729/jnma.4789

**Published:** 2020-01-31

**Authors:** Madhu Rajeshwari, Pirabu Sakthivel, Rijendra Yogal, Smriti Panda, Chirom Amit Singh, Deepali Jain

**Affiliations:** 1 Department of Pathology, All India Institute of Medical Sciences, New Delhi, India; 2 Department of Otorhinolaryngology and Head-Neck surgery, All India Institute of Medical Sciences, New Delhi, India

**Keywords:** *combined small cell carcinoma*, *composite tumor*, *larynx*, *small cell carcinoma*, *squamous cell carcinoma*

## Abstract

Composite tumor of larynx, a recently included entity in the current WHO classification, is often a difficult pathological diagnosis, especially in small biopsies. We report a case of laryngeal composite tumor, initially misdiagnosed as squamous cell carcinoma, which later turned out to be composite in nature, with associated neuroendocrine (small cell carcinoma) component. This report emphasizes the need for obtaining deeper biopsies and their thorough pathological examination to improve the diagnostic accuracy.

## INTRODUCTION

Laryngeal carcinoma accounts for approximately 2% to 5% of new malignancies worldwide every year, of which 85% to 95 % are squamous cell carcinomas.^1,2^ Neuroendocrine tumors (NET) of the larynx account for less than 0.5% of all laryngeal tumors, the most common being atypical carcinoid followed by small cell carcinoma.^3^ Small cell carcinomas associated with a squamous or adenocarcinoma component are referred to as combined small cell carcinomas.

Laryngeal localization of combined tumors is an extremely rare occurrence with only 20 cases reported in the English literature till date. Combined small cell carcinoma is an uncommon aggressive laryngeal malignancy. Both the components may not be represented in small biopsies, making preoperative diagnosis difficult. Specific diagnosis of both the components of combined carcinomas is of paramount importance as treatment depends on diagnostic accuracy. These tumors are now recognized as a distinct entity in the current WHO classification of head and neck tumors.^1^ Due to their rarity, there is a lack of information regarding the natural history, treatment and prognosis of these patients.

Here in, we report a case of combined small cell carcinoma which was initially diagnosed as primary SCC of the larynx and had an initial complete response. However, the disease relapsed within 2 months after therapy when the biopsy revealed the composite nature of the tumor with an additional small cell neuroendocrine carcinoma component, leading to a fatal outcome.

## CASE REPORT:

**Figure 1.**
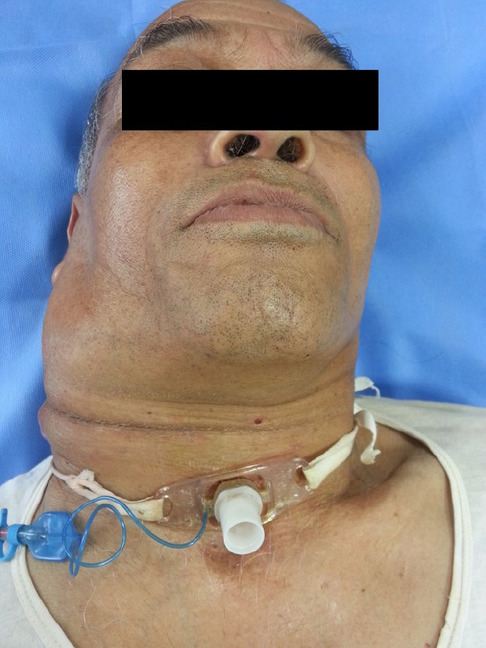
Clinical picture after extensive locoregional recurrence.

A 50-year-old male was initially diagnosed as a case of squamous cell carcinoma larynx, stage T4aN2bM0 at a health care center elsewhere. He was advised surgery however, the patient refused and underwent organ-preserving concomitant chemo-radiotherapy. After six cycles of cisplatin-based chemotherapy and 35 fractions/70gy of radiotherapy, the patient achieved complete remission. After a 2-month disease-free interval, he developed rapidly enlarging bilateral neck swellings along with progressive hoarseness and respiratory distress (Figure 1), for which he underwent an emergency tracheostomy. The patient was referred to our center for further management. Endoscopy revealed a large ulcerative growth involving right supraglottis with edematous endolarynx causing airway compromise [rT4aN3bM0].

**Figure 2.**
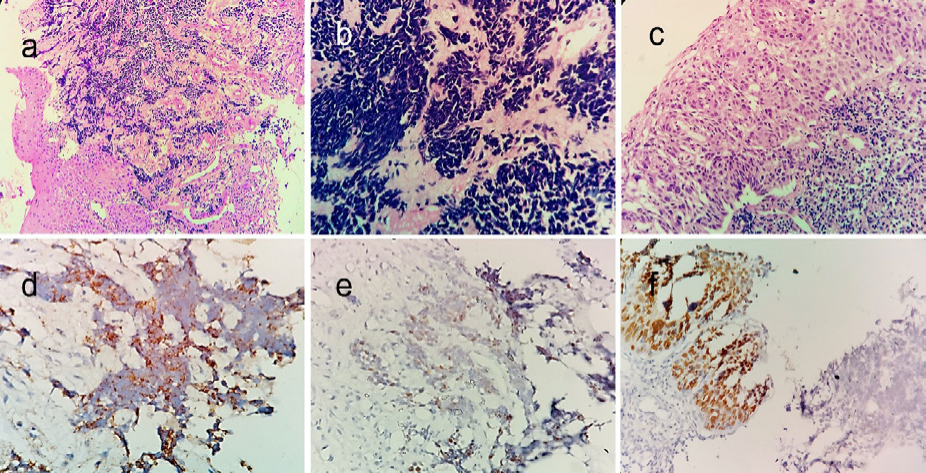
Photomicrograph showing subepithelial nests of tumor cells with features of small cell carcinoma (a, H&E, ×100). Tumor cells have scant cytoplasm and hyperchromatic nuclei with prominent nuclear moulding and crush artefact (b, H&E, ×400). Overlying squamous epithelium displaying full thickness dysplasia (carcinoma in situ) (c, H&E, ×200). Small cell component is immunopositive for synaptophysin (d) and TTF-1 (e), while squamous component is immunopositive for p40 (f) (IHC, ×400).

A repeat biopsy from right supraglottis revealed a predominant small cell carcinoma component, immunopositive for synaptophysin and CD56, and immunonegative for p40. A small focus of squamous cell carcinoma in situ (highlighted by p40 immunostain) was also noted (Figure 2).

**Figure 3.**
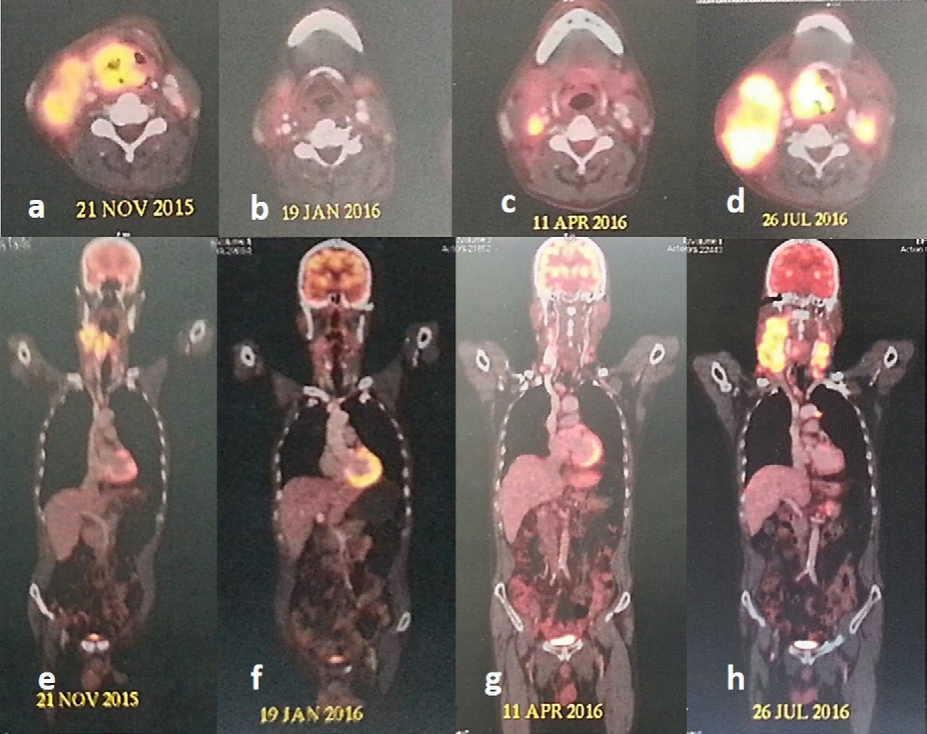
Serial whole-body PET-CT scans demonstrating the evolution of disease.

A final diagnosis of combined small cell carcinoma was rendered. A whole-body PET scan revealed a large volume loco-regional progression of the tumor (Figure 3) without any evidence of distant metastasis.

Due to the poor general condition of the patient and extensive loco-regional relapse, the patient was treated with palliative metronomic chemotherapy (weekly Tab. methotrexate 20mg once weekly) for 3 months, after which he died of progressive disease.

## DISCUSSION

Combined small cell carcinoma, as defined by the International Association for the Study of Lung Cancer, refers to small cell carcinoma with a component of squamous cell or adenocarcinoma. Ferlito et al first described the term “Combined small cell carcinomas of larynx” and Gnepp et al used the term “Composite tumor of larynx” to describe these morphologically distinct tumors showing divergent lineages of differentiation at the same site.^2,3^ Rosa et al in 2016 employed the term “mixed neuroendocrine-non-neuroendocrine neoplasm (MiNEN)” to unify the concept of these morphologically heterogeneous group of neoplasms, which show different traits in their clinical behavior, thereby demanding distinctive treatment strategies for appropriate management of the neuroendocrine and non-neuroendocrine components.^1^

In general, the histogenesis of these tumors may be explained by “collision theory” i.e. the result of the combined growth of two different neoplastic clones or by “common precursor theory” i.e. the proliferation of a common precursor cell with divergent differentiation. The latter is supported by the presence of the morphological spectrum of transition between the two components and by various molecular studies that have demonstrated identical genetic alterations in both the components.^7^ Hence the speculation that these biphasic neoplasms are derived from a common neoplastic stem cell that can differentiate into the Kulchitsky cells (neuroendocrine cells) as well as squamous or glandular cells in the appropriate microenvironment seems more plausible.

Review of the previously reported cases of composite tumors of larynx revealed the non-neuroendocrine component to be squamous cell carcinoma in all the cases, while the neuroendocrine component was small cell carcinoma in all but one case which was an atypical carcinoid.^2,3,5–15^ Males in their fifth and sixth decades were affected predominantly [range 32-83 years; median 55]. Smoking was a risk factor in all the patients, except for one. None of the cases had any associated paraneoplastic syndromes. Supraglottis was the most common site involved. An initial biopsy was available in 11 cases, among which only three cases had both the components, thereby rendering a correct preoperative diagnosis. In one of the cases, the squamous component was represented only by an in situ lesion, similar to our case. Almost all patients received multimodality treatment. In fact, of the 18 patients in which the outcome was known, 8 died for the disease after a mean follow-up of 19 months, whereas 4 were alive with the disease after a mean follow-up of 24 months and the remaining 6 did not have evidence of disease after a mean follow-up of 26 months.

Interestingly, in two of the cases, there was a lateralization of individual tumor components on either side of the larynx. Even more curious was the fact that the side specificity of the components was reproduced in the metastatic nodal spread in one of these cases.^14^

Preoperative accurate histopathological diagnosis is of utmost importance to ensure optimal treatment and often represents a pathological challenge. Since many times the squamous or the small cell component may be only seen in preoperative biopsy samples for which the treatment entirely differs and results in faulty treatment. The pre-operative diagnostic yield of small cell carcinoma (pure or combined) can be enhanced by including deeper submucosal biopsies on laryngoscopy in all those cases in which the extent of disease on imaging is disproportionately larger than the apparent mucosal involvement on laryngoscopy.^14^ Due to the rare possibility of lateralization of tumor components, extensive sampling should be done in bilateral laryngeal tumors or neck nodes.

Histologically, combined small cell carcinomas are similar to their pulmonary counterparts. The small cell carcinoma is represented by small cells with scant cytoplasm, hyperchromatic nuclei, and inconspicuous nucleoli. Brisk mitoses, necrosis and crush artifact are also present. Tumor cells exhibit variable immunopositivity for neuroendocrine markers like synaptophysin, chromogranin, and CD56. TTF1 may be positive. MIB1 labeling index is very high. On the other hand, the cells of squamous cell carcinoma are larger with moderate cytoplasm, vesicular nucleus, conspicuous nucleolus and exhibit varying degrees of keratinization. These cells will be immunopositive for p40, p63 and high molecular weight cytokeratin.^3,4^

Before rendering a diagnosis of a composite tumor of the larynx, it is important to ascertain by a thorough clinical workup that the small cell carcinoma component is not a metastasis from either gastrointestinal or bronchogenic sites. Inspite of the rarity of these tumors, the clinical course of the composite is similar to that of small cell carcinoma of the larynx as it displays aggressive biologic behavior with early distant metastasis.^1,11^ Composite tumor of the larynx constitutes a real challenge for clinicians due to the initial difficulty in correct diagnosis and the need for integrated treatment modalities. Pure SCC of the larynx is best treated with either surgery and/or radio-chemotherapy. On the contrary, for SmCC of the larynx, due to its early metastatic potential, it is advisable to administer systemic chemotherapy rather than primary unimodality therapy, such as radiotherapy or surgery alone. Commonly used agents include cisplatin, carboplatin, etoposide, cyclophosphamide, doxorubicin, vincristine, and methotrexate.^12^ Combined small cell carcinoma warrants a multimodal treatment regime. Whenever a small cell carcinoma component is identified in combined tumors, radiotherapy should be the first line of treatment. Chemotherapy must also be incorporated early in the treatment irrespective of the volume of the small cell component. Surgery may be reserved for cases with local relapse or persistent disease after chemo-radiotherapy. However, despite aggressive treatment patients may have an early relapse and the prognosis is relatively poor, with two and five-year survival rates of only 16 and 5 percent, respectively.^12,16^

For atypical carcinoids, surgery is the primary treatment as radiotherapy and chemotherapy are ineffective.^13^ Overall survival rates of 48 percent at five years and 30 percent at 10 years have been reported.^17^

## Consent:

**JNMA Case Report Consent Form** was signed by the patient and the original is attached with the patient chart.

## Conflict of Interest

**None.**
